# Endophytic *Pestalotiopsis* species associated with *Rhododendron* in Cangshan Mountain, Yunnan Province, China

**DOI:** 10.3389/fmicb.2022.1016782

**Published:** 2022-10-20

**Authors:** Rui Gu, Dan-Feng Bao, Hong-Wei Shen, Xi-Jun Su, Yun-Xia Li, Zong-Long Luo

**Affiliations:** ^1^College of Agronomy and Biosciences, Dali University, Dali, Yunnan, China; ^2^Center of Excellence in Fungal Research, Mae Fah Luang University, Chiang Rai, Thailand; ^3^Department of Entomology and Plant Pathology, Faculty of Agriculture, Chiang Mai University, Chiang Mai, Thailand; ^4^School of Science, Mae Fah Luang University, Chiang Rai, Thailand

**Keywords:** six new species, endophytic fungi, Sordariomycetes, morphology, phylogeny

## Abstract

*Rhododendron* is an essential ornamental plant that is abundant in Yunnan province. In Cangshan Mountain, Yunnan, China, 61 species of *Rhododendron* have been reported. Endophytic fungi are internal plant tissue inhabitants that do not harm the host. It has emerged as an exciting research topic as they have the potential to provide numerous secondary metabolites. This study is focused on taxonomic novelties and new host records of endophytic fungi associated with *Rhododendron* plants collected from Cangshan Mountain in Yunnan Province, China. *Pestalotiopsis* fungi are associated with a vast array of plant species worldwide. In this study, fresh leaves of *Rhododendron cyanocarpum*, *Rhododendron decorum*, and *Rhododendron delavayi* were collected from Cangshan Mountain, Yunnan Province, China. Endophytic *Pestalotiopsis* fungi associated with *Rhododendron* were characterized based on phylogenetic analyses of combined ITS, TEF1-α, and TUB genes along with morphological characteristics. Six new species (*Pestalotiopsis appendiculata*, *Pestalotiopsis cangshanensis*, *Pestalotiopsis daliensis*, *Pestalotiopsis fusoidea*, *Pestalotiopsis rosarioides*, and *Pestalotiopsis suae*) and a new host record (*Pestalotiopsis trachicarpicola*) are described. Detailed descriptions and color photo plates of these species are provided. It is the first time that the endophytic fungi of *Rhododendron* plants in Cangshan Mountain have been studied.

## Introduction

*Rhododendron* is the largest genus of woody plants in the Northern Hemisphere and the largest genus in *Ericaceae* (Fang et al., 2005; Shrestha et al., 2018). *Rhododendron* is an important component of montane ecosystems, with more than 1,025 species and approximately 581 species in China (Cai et al., 2016; Ma et al., 2017; Cao et al., 2022). Moreover, Yunnan province in southwest China is a center of diversity for *Rhododendron* (Ma et al., 2021). *Rhododendron* is a popular landscape plant and a food source (Negi et al., 2013; Lin et al., 2021). Due to the economic significance of this plant, it is essential to evaluate the fungi associated with it (Chaiwan et al., 2022). Pathogenic fungi, mycorrhizal fungi, and endophytic fungi have been isolated from *Rhododendron* in previous research (Zhang et al., 2019); however, there is no record of endophytic fungi associated with *Rhododendron* in Cangshan Mountain, Yunnan, China.

Endophytic fungi or endophytes exist widely inside the healthy tissues of living plants and are important components of plant micro-ecosystems (Jia et al., 2016). Endophytic fungi benefit their host plants by increasing their drought resistance, disease resistance, and growth-promoting properties (Rodriguez et al., 2009; De Silva et al., 2019; Rashmi et al., 2019). Endophytic fungi have the potential to produce metabolites with a wide range of biological activities, making them an appealing research topic (Huang et al., 2009; De Silva et al., 2019; Rashmi et al., 2019). More than 800 endophytic fungal genera have been reported worldwide, most speciose genera (>50 species) are *Penicillium* (103), *Colletotrichum* (78), *Alternaria* (61), *Fusarium* (59), *Pestalotiopsis* (53), and *Aspergillus* (52) (Rashmi et al., 2019). Amongst the different substrates, leaf endophytes have been studied and analyzed in more detail when compared to other parts (Rashmi et al., 2019).

Steyaert (1949) introduced *Pestalotiopsis* to accommodate species with fusiform conidia with three colored median cells and two colorless end cells, as well as two or more apical appendages. Traditionally, taxonomy and identification of *Pestalotiopsis* and allied genera were based mainly on conidial characters (Steyaert, 1949; Guba, 1961) and conidiogenesis (Sutton, 1980). Hu et al. (2007) reported that conidial characteristics, such as conidial length, median cell length, conidial width, and median cell color, were insignificantly different within *Pestalotiopsis*. Maharachchikumbura et al. (2014) selected internal transcribed spacer (ITS), partial β-tubulin (TUB), and partial translation elongation factor 1-alpha (TEF1-α), along with morphological characters to resolve the identification of *Pestalotiopsis*. Up to now, in a total of 92 *Pestalotiopsis* species have been introduced based on morphological and phylogenetic evidence (Maharachchikumbura et al., 2014; Liu et al., 2019; Rashmi et al., 2019; Shu et al., 2020; Monteiro et al., 2022). *Pestalotiopsis* is ordinarily isolable as endophytes in plants (Aly et al., 2010; Watanabe et al., 2010; Maharachchikumbura et al., 2012). However, there is only one *Pestalotiopsis* species viz., *Pestalotiopsis baarnensis* associated with *Rhododendron* (Rashmi et al., 2019).

As a part of the investigation on endophytic fungal diversity associated with *Rhododendron* plants in Cangshan Mountain, Yunnan province, China, we revealed seven *Pestalotiopsis*-like taxa from fresh leaves of *Rhododendron*. Their taxonomic positions were established based on morphological descriptions and multi-locus phylogenetic analyses. The endophytic fungal strain resources were stored for future study on their secondary metabolites.

## Materials and methods

### Isolation and morphology

Fresh *Rhododendron* (Ericaceae) leaves were obtained from Cangshan Mountain, Yunnan Province, China. The gathered leaves were placed in a sterile polyethylene bag and stored at 4°C. The symptomless leaves of each *Rhododendron* spp. were treated with gently running tap water to remove the surface debris. They were surface-sterilized by using 75% ethanol for 1 min, 0.1% HgCl_2_ for 3 min, and washed five times using sterile distilled water, finally dried on sterile filter paper (Tao et al., 2013). The 5-mm diameter leaf discs treated as above were placed on potato dextrose agar (PDA) plates without antibiotics. The PDA plates were incubated in ambient light at 25°C. When colonies appeared, they were transferred onto new PDA plates and further incubated in ambient light at 25°C for morphological examination. Sporulation was induced on pine needle medium (“pine needle” and 1/10-strength PDA). Macromorphological characters of conidiomata on PDA were observed using an Optec SZ 760 compound stereomicroscope. Temporarily prepared microscope slides were placed under a Nikon ECLIPSE Ni-U compound stereomicroscope for observation and micro-morphological photography. Part of the pure culture that produced spores was removed and put into a water–agar medium (WA) with glycerol and air-dried at room temperature (De Silva et al., 2019). All endophytic isolates are stored at the Culture Collection of Kunming Institute of Botany, the Chinese Academy of Sciences (KUNCC), and the China General Microbiological Culture Collection Center (CGMCC). The Herbarium of Cryptogams Kunming Institute of Botany Academia Sinica (Herb. KUN-HKAS) housed the herbarium specimens. The MycoBank^1^ number was registered (Crous et al., 2004).

### DNA extraction, PCR amplification, and sequencing

Genomic DNA extraction was carried out from fresh mycelium growing on PDA at 25°C using the Trelief™ Plant Genomic DNA Kit according to the manufacturer’s instructions. The primer pairs ITS5/ITS4, EF1-728F/EF2, and Bt2a/Bt2b were used to amplify the ITS, TEF1-α, and TUB gene regions, respectively. The amplification was performed in a 25 μL reaction volume containing 12.5 μL of Master Mix (Tsingke Biotech, Yunnan, China), 1 μL of each primer (10 μm), 1 μL of template DNA, and 9.5 μL of deionized water. The PCR thermal cycles for three genes were performed under the following reaction conditions: an initial denaturing step for 94°C for 3 min followed by 35 cycles of denaturation at 94°C for 45 s, annealing at 55°C for 45 s for ITS and TEF1-α, and 56°C for 60 s for TUB, elongation at 72°C for 1 min, and a final extension at 72°C for 10 min. PCR products were verified on 1% agarose electrophoresis gels stained with ethidium bromide. Sequencing was carried out by Tsingke Biological Engineering Technology and Services Co., Ltd. (Yunnan, China).

### Molecular phylogenetic analyses

#### Sequence alignment

Sequences with high similarity indices were assembled in BioEdit, and those with low similarity indices were identified through a BLAST search for the closest matches with *Pestalotiopsis* taxa and from recently published data (Li et al., 2021). All consensus and reference sequences were automatically aligned with MAFFT v.7 using the Auto strategy (Katoh and Standley, 2013). The aligned sequences from each gene region (ITS, TEF1-α, and TUB) were combined and manually improved using Sequence Matrix (Hall, 1999). Uncertain regions were omitted from the alignment, and gaps were treated as data that was missing. Maximum likelihood (ML) and Bayesian inference were used to conduct phylogenetic analyses.

#### Phylogenetic analyses

Maximum likelihood analysis was performed at the CIPRES Science Gateway v.3.3 (Miller et al., 2010) using RAxML v.8.2.8 as part of the “RAxML-HPC2 on XSEDE” tool (Stamatakis, 2006; Stamatakis et al., 2008). The optimal ML tree search was conducted with 1,000 separate runs using the default algorithm of the programme from a random starting tree for each run. The final tree was selected amongst suboptimal trees from each run by comparing the likelihood scores using the GTR+GAMMA substitution model. Maximum likelihood bootstrap values equal to or greater than 60% were given as the first set of numbers above the nodes in the resulting ML tree.

Bayesian analysis was performed with MrBayes v.3.1.2 (Ronquist and Huelsenbeck, 2003) to evaluate posterior probabilities (Rannala and Yang, 1996) using Markov Chain Monte Carlo sampling (MCMC). The best-fit model of evolution was estimated using MrModeltest v.2.2 (Nylander, 2004). For Bayesian analysis, the best-fitting model of ITS, TEF1-α, and TUB was the GTR+I+G model. Posterior probabilities (PPs) (Rannala and Yang, 1996) were performed using Markov chain Monte Carlo sampling (BMCMC) in MrBayes v.3.1.2 (Liu et al., 2012). Six concurrent Markov chains were executed for 50 million generations, and samples of trees were taken every 5,000 generations (resulting in 10,000 trees). The initial 2,000 trees representing the burn-in phase of the analyses were discarded, while the remaining 8,000 trees were used to calculate PP in the majority rule consensus tree (Cai et al., 2006; Liu et al., 2012).

Phylogenetic trees were displayed in FigTree v. 1.4.4 (Rambaut, 2014) and edited in Adobe Illustrator CS5 (Adobe Systems, San Jose, CA, USA). Newly generated sequences were deposited in GenBank (Table 1).

**TABLE 1:** GenBank numbers and culture collection accession numbers of species included in the phylogenetic study.

Taxa	Strain	GenBank accession no.			References
		ITS	TEF1-α	TUB	
*Pestalotiopsis adusta*	ICMP 6088	AF409957	JX399070	JX399037	Maharachchikumbura et al., 2012
*Pestalotiopsis adusta*	MFLUCC 10–0146	JX399007	JX399071	JX399038	Maharachchikumbura et al., 2012
*Pestalotiopsis aggestorum*	LC6301	KX895015	KX895234	KX895348	Liu et al., 2017
*Pestalotiopsis aggestorum*	LC8186	KY464140	KY464150	KY464160	Liu et al., 2017
*Pestalotiopsis anacardiacearum*	IFRDCC 2397	KC247154	KC247156	KC247155	Maharachchikumbura et al., 2013
*Pestalotiopsis anacardiacearum*	HN37–4	-	MK512485	MK360932	Shu et al., 2020
*Pestalotiopsis anacardiacearum*	YB41–2	-	MK512486	MK360933	Shu et al., 2020
*Pestalotiopsis anacardiacearum*	FY10–12	-	MK512484	MK360931	Shu et al., 2020
***Pestalotiopsis appendiculata***	**CGMCC 3.23550**	**OP082431**	**OP185509**	**OP185516**	**This study**
*Pestalotiopsis arceuthobii*	CBS 434.65	NR147561	KM199516	KM199427	Maharachchikumbura et al., 2014
*Pestalotiopsis arengae*	CBS 331.92	NR147560	KM199515	KM199426	Maharachchikumbura et al., 2014
*Pestalotiopsis australasiae*	CBS 114126	NR147546	KM199499	KM199409	Maharachchikumbura et al., 2014
*Pestalotiopsis australasiae*	CBS 114141	KM199298	KM199501	KM199410	Maharachchikumbura et al., 2014
*Pestalotiopsis australis*	CBS 111503	KM199331	KM199557	KM199382	Maharachchikumbura et al., 2014
*Pestalotiopsis australis*	CBS 114193	KM199332	KM199475	KM199383	Maharachchikumbura et al., 2014
*Pestalotiopsis biciliata*	CBS 124463	KM199308	KM199505	KM199399	Maharachchikumbura et al., 2014
*Pestalotiopsis biciliata*	CBS 236.38	KM199309	KM199506	KM199401	Maharachchikumbura et al., 2014
*Pestalotiopsis biciliata*	CBS 790.68	KM199305	KM199507	KM199400	Maharachchikumbura et al., 2014
*Pestalotiopsis brachiata*	LC2988	KX894933	KX895150	KX895265	Liu et al., 2017
*Pestalotiopsis brachiata*	LC8188	KY464142	KY464152	KY464162	Liu et al., 2017
*Pestalotiopsis brassicae*	CBS 170.26	KM199379	KM199558	-	Maharachchikumbura et al., 2014
*Pestalotiopsis camelliae*	CBS 443.62	KM199336	KM199512	KM199424	Maharachchikumbura et al., 2014
*Pestalotiopsis camelliae*	MFLUCC 12–0277	NR120188	JX399074	JX399041	Zhang et al., 2012a
*Pestalotiopsis camelliae-oleiferae*	LHLKD 08	OK493593	OK507963	OK562368	Li et al., 2021
*Pestalotiopsis camelliae-oleiferae*	LHLKD 09	OK493594	OK507964	OK562369	Li et al., 2021
*Pestalotiopsis camelliae-oleiferae*	LHLKD 10	OK493595	OK507965	OK562370	Li et al., 2021
***Pestalotiopsis cangshanensis***	**CGMCC 3.23544**	**OP082426**	**OP185510**	**OP185517**	**This study**
*Pestalotiopsis chamaeropis*	CBS 113607	KM199325	KM199472	KM199390	Maharachchikumbura et al., 2014
*Pestalotiopsis chamaeropis*	CBS 186.71	KM199326	KM199473	KM199391	Maharachchikumbura et al., 2014
*Pestalotiopsis clavata*	MFLUCC 12–0268	JX398990	JX399056	JX399025	Maharachchikumbura et al., 2012
*Pestalotiopsis colombiensis*	CBS 118553	NR147551	KM199488	KM199421	Maharachchikumbura et al., 2014
***Pestalotiopsis daliensis***	**CGMCC 3.23548**	**OP082429**	**OP185511**	**OP185518**	**This study**
*Pestalotiopsis digitalis*	ICMP 5434	KP781879	-	KP781883	Maharachchikumbura et al., 2016
*Pestalotiopsis diploclisiae*	CBS 115585	KM199315	KM199483	KM199417	Maharachchikumbura et al., 2014
*Pestalotiopsis diploclisiae*	CBS 115587	KM199320	KM199486	KM199419	Maharachchikumbura et al., 2014
*Pestalotiopsis diploclisiae*	CBS 115449	KM199314	KM199485	KM199416	Maharachchikumbura et al., 2014
*Pestalotiopsis disseminata*	CBS 118552	MH553986	MH554410	MH554652	Liu et al., 2019
*Pestalotiopsis disseminata*	CBS 143904	MH554152	MH554587	MH554825	Liu et al., 2019
*Pestalotiopsis disseminata*	CPC 29351	MH554166	MH554601	MH554839	Liu et al., 2019
*Pestalotiopsis distincta*	LC3232	KX894961	KX895178	KX895293	Liu et al., 2017
*Pestalotiopsis distincta*	LC8184	KY464138	KY464148	KY464158	Liu et al., 2017
*Pestalotiopsis diversiseta*	MFLUCC 12–0287	JX399009	JX399073	JX399040	Maharachchikumbura et al., 2012
*Pestalotiopsis doitungensis*	MFLUCC 14–0090	MK993573	MK975831	MK975836	Ma et al., 2019
*Pestalotiopsis dracaenae*	HGUP4037	MT596515	MT598644	MT598645	Ariyawansa et al., 2015
*Pestalotiopsis dracaenicola*	MFLUCC 18–0913	MN962731	-	-	Chaiwan et al., 2020
*Pestalotiopsis dracaenicola*	MFLUCC 18–0914	MN962734	-	-	Chaiwan et al., 2020
*Pestalotiopsis dracontomelon*	MFLUCC 10–0149	KP781877	KP781880	-	Maharachchikumbura et al., 2016
*Pestalotiopsis endophytic*	MFLUCC 18–0932	NR 172439	MW417119	-	De Silva et al., 2021
*Pestalotiopsis endophytic*	MFLUCC 20–0142	MW263948	-	-	De Silva et al., 2021
*Pestalotiopsis endophytic*	MFLUCC 18–0946	MW263947	MW729384	-	De Silva et al., 2021
*Pestalotiopsis ericacearum*	IFRDCC 2439	KC537807	KC53784	KC537821	Zhang et al., 2013
*Pestalotiopsis etonensis*	BRIP 66615	MK966339	MK97765	MK977634	Crous et al., 2020
*Pestalotiopsis formosana*	NTUCC 17–009	MH809381	MH809389	MH809385	Ariyawansa et al., 2015
*Pestalotiopsis formosana*	NTUCC 17–010	MH809382	MH809390	MH809386	Ariyawansa et al., 2015
*Pestalotiopsis furcata*	LC6303	KX895016	KX895235	KX895349	Liu et al., 2017
*Pestalotiopsis furcata*	MFLUCC 12–0054	JQ683724	JQ683740	JQ683708	Maharachchikumbura et al., 2013
***Pestalotiopsis fusoidea***	**CGMCC 3.23545**	**OP082427**	**OP185512**	**OP185519**	**This study**
*Pestalotiopsis gaultheri*	IFRD 411–014	KC537805	KC537812	KC537819	Maharachchikumbura et al., 2014
*Pestalotiopsis gibbosa*	NOF 3175	LC311589	LC311591	LC311590	Watanabe et al., 2018
*Pestalotiopsis grevilleae*	CBS 114127	KM199300	KM199504	KM199407	Maharachchikumbura et al., 2014
*Pestalotiopsis hawaiiensis*	CBS 114491	NR147559	KM199514	KM199428	Maharachchikumbura et al., 2014
*Pestalotiopsis hispanica*	CBS 115391	MH553981	MH554399	MH554640	Liu et al., 2019
*Pestalotiopsis hollandica*	CBS 265.33	NR147555	KM199481	KM199388	Maharachchikumbura et al., 2014
*Pestalotiopsis humus*	CBS 336.97	KM199317	KM199484	KM199420	Maharachchikumbura et al., 2014
*Pestalotiopsis hunanensis*	LHXT 15	OK493599	OK507969	OK562374	Li et al., 2021
*Pestalotiopsis hunanensis*	LHXT 18	OK493600	OK507970	OK562375	Li et al., 2021
*Pestalotiopsis hunanensis*	LHXT 19	OK493601	OK507971	OK562376	Li et al., 2021
*Pestalotiopsis hydei*	MFLUCC 20–0135	NR 172003	MW251113	MW251112	Huanaluek et al., 2021
*Pestalotiopsis iberica*	CAA 1004	MW732250	MW759038	MW759034	Monteiro et al., 2022
*Pestalotiopsis iberica*	CAA 1005	MW732248	MW759037	MW759035	Monteiro et al., 2022
*Pestalotiopsis iberica*	CAA 1006	MW732249	MW759039	MW759036	Monteiro et al., 2022
*Pestalotiopsis inflexa*	MFLUCC 12–0270	JX399008	JX399072	JX399039	Maharachchikumbura et al., 2012
*Pestalotiopsis intermedia*	MFLUCC 12–0259	JX398993	JX399059	JX399028	Maharachchikumbura et al., 2012
*Pestalotiopsis italiana*	MFLUCC 12–0657	KP781878	KP781881	KP781882	Liu et al., 2015
*Pestalotiopsis jesteri*	CBS 109350	KM199380	KM199554	KM199468	Maharachchikumbura et al., 2014
*Pestalotiopsis jiangxiensis*	LC4399	KX895009	KX895227	KX895341	Liu et al., 2017
*Pestalotiopsis jinchanghensis*	LC6636	KX895028	KX895247	KX895361	Liu et al., 2017
*Pestalotiopsis jinchanghensis*	LC8190	KY464144	KY464154	KY464164	Liu et al., 2017
*Pestalotiopsis kandelicola*	NCYUCC 19–0355	MT560722	MT563101	MT563099	Hyde et al., 2020
*Pestalotiopsis kandelicola*	NCYUCC 19–0354	MT560723	MT563102	MT563100	Hyde et al., 2020
*Pestalotiopsis kaki*	KNU-PT-1804	LC552953	LC553555	LC552954	Das et al., 2020
*Pestalotiopsis kenyana*	CBS 442.67	KM199302	KM199502	KM199395	Maharachchikumbura et al., 2014
*Pestalotiopsis krabiensis*	MFLUCC 16–0260	MH388360	MH388395	MH412722	Tibpromma et al., 2018
*Pestalotiopsis knightiae*	CBS 114138	KM199310	KM199497	KM199408	Maharachchikumbura et al., 2014
*Pestalotiopsis knightiae*	CBS 111963	KM199311	KM199495	KM199406	Maharachchikumbura et al., 2014
*Pestalotiopsis leucadendri*	CBS 121417	MH553987	MH554412	MH554654	Liu et al., 2019
*Pestalotiopsis licualacola*	HGUP 4057	KC492509	KC481684	KC481683	Geng et al., 2013
*Pestalotiopsis linearis*	MFLUCC 12–0271	JX398994	JX399060	JX399027	Maharachchikumbura et al., 2012
*Pestalotiopsis lushanensis*	LC4344	KX895005	KX895223	KX895337	Liu et al., 2017
*Pestalotiopsis lushanensis*	LC8182	KY464136	KY464146	KY464156	Liu et al., 2017
*Pestalotiopsis macadamiae*	BRIP 63738b	KX186588	KX186620	KX186680	Akinsanmi et al., 2017
*Pestalotiopsis malayana*	CBS 102220	NR147550	KM199482	KM199411	Maharachchikumbura et al., 2014
*Pestalotiopsis monochaeta*	CBS 144.97	KM199327	KM199479	KM199386	Maharachchikumbura et al., 2014
*Pestalotiopsis monochaeta*	CBS 440.83	KM199329	KM199480	KM199387	Maharachchikumbura et al., 2014
*Pestalotiopsis montellica*	MFLUCC 12–0279	JX399012	JX399076	JX399043	Maharachchikumbura et al., 2012
*Pestalotiopsis nanjingensis*	LHNJ 16	OK493602	OK507972	OK562377	Li et al., 2021
*Pestalotiopsis nanjingensis*	LHNJ 20	OK493603	OK507973	OK562378	Li et al., 2021
*Pestalotiopsis nanjingensis*	LHNJ 04	OK493604	OK507974	OK562379	Li et al., 2021
*Pestalotiopsis nanningensis*	LHGX 10	OK493596	OK507966	OK562371	Li et al., 2021
*Pestalotiopsis nanningensis*	LHGX 11	OK493597	OK507967	OK562372	Li et al., 2021
*Pestalotiopsis nanningensis*	LHGX 12	OK493598	OK507968	OK562373	Li et al., 2021
*Pestalotiopsis neglecta*	TAP1100	AB482220	LC311600	LC311599	Watanabe et al., 2018
*Pestalotiopsis neolitseae*	NTUCC 17–011	MH809383	MH809391	MH809387	Ariyawansa and Hyde, 2018
*Pestalotiopsis neolitseae*	NTUCC 17–012	MH809384	MH809392	MH809388	Ariyawansa and Hyde, 2018
*Pestalotiopsis neolitseae*	KUMCC 19–0243	MN625276	MN626741	MN626730	Ariyawansa and Hyde, 2018
*Pestalotiopsis novae-hollandiae*	CBS 130973	NR147557	KM199511	KM199425	Maharachchikumbura et al., 2014
*Pestalotiopsis oryzae*	CBS 111522	KM199294	KM199493	KM199394	Maharachchikumbura et al., 2014
*Pestalotiopsis oryzae*	CBS 353.69	KM199299	KM199496	KM199398	Maharachchikumbura et al., 2014
*Pestalotiopsis pallidotheae*	MAFF 240993	NR111022	LC311585	LC311584	Watanabe et al., 2010
*Pestalotiopsis pandanicola*	MFLUCC 16–0255	MH388361	MH388396	MH412723	Tibpromma et al., 2018
*Pestalotiopsis papuana*	CBS 331.96	KM199321	KM199491	KM199413	Maharachchikumbura et al., 2014
*Pestalotiopsis papuana*	CBS 887.96	KM199318	KM199492	KM199415	Maharachchikumbura et al., 2014
*Pestalotiopsis papuana*	MFLU 19–2764	-	MW192204	MW296942	Maharachchikumbura et al., 2014
*Pestalotiopsis parva*	CBS 265.37	KM199312	KM199508	KM199404	Maharachchikumbura et al., 2014
*Pestalotiopsis parva*	CBS 278.35	MH855675	KM199509	KM199405	Maharachchikumbura et al., 2014
*Pestalotiopsis photinicola*	GZCC 16–0028	KY092404	KY047662	KY047663	Chen et al., 2017
*Pestalotiopsis pini*	CBS 146841	MT374681	MT374694	MT374706	Silva et al., 2020
*Pestalotiopsis pini*	CBS 146840	MT374680	MT374693	MT374705	Silva et al., 2020
*Pestalotiopsis pini*	CBS 146842	MT374682	MT374695	MT374707	Silva et al., 2020
*Pestalotiopsis pini*	MEAN 1167	MT374689	MT374701	MT374714	Silva et al., 2020
*Pestalotiopsis pinicola*	KUMCC 19–0203	MN412637	MN417510	MN417508	Tibpromma et al., 2019
*Pestalotiopsis pinicola*	KUMCC 19–0183	MN412636	MN417509	MN417507	Tibpromma et al., 2019
*Pestalotiopsis portugalica*	CBS 393.48	KM199335	KM199510	KM199422	Maharachchikumbura et al., 2014
*Pestalotiopsis portugalica*	LC2929	KX894921	KX895138	KX895253	Liu et al., 2017
*Pestalotiopsis rhizophorae*	MFLUCC 17–0416	MK764283	MK764327	MK764349	Norphanphoun et al., 2019
*Pestalotiopsis rhizophorae*	MFLUCC 17–0417	MK764284	MK764328	MK764350	Norphanphoun et al., 2019
*Pestalotiopsis rhododendri*	OP086	KC537804	KC537811	KC537818	Zhang et al., 2013
*Pestalotiopsis rhodomyrtus*	LC3413	KX894981	KX895198	KX895313	Liu et al., 2017
*Pestalotiopsis rhodomyrtus*	LC4458	KX895010	KX895228	KX895342	Liu et al., 2017
*Pestalotiopsis rosea*	MFLUCC 12–0258	JX399005	JX399069	JX399036	Maharachchikumbura et al., 2012
***Pestalotiopsis rosarioides***	**CGMCC 3.23549**	**OP082430**	**OP185513**	**OP185520**	**This study**
*Pestalotiopsis scoparia*	CBS 176.25	KM199330	KM199478	KM199393	Maharachchikumbura et al., 2014
*Pestalotiopsis sequoiae*	MFLUCC 13–0399	KX572339	-	-	Hyde et al., 2016
*Pestalotiopsis shandongensis*	KUMCC 19 0241	MN625275	MN626740	MN626729	Maharachchikumbura et al., 2014
*Pestalotiopsis shorea*	MFLUCC 12–0314	KJ503811	KJ503817	KJ503814	Song et al., 2014
*Pestalotiopsis spathulata*	CBS 356.86	NR147558	KM199513	KM199423	Maharachchikumbura et al., 2014
*Pestalotiopsis spathuliappendiculata*	CBS 144035	MH554172	MH554607	MH554845	Liu et al., 2019
***Pestalotiopsis suae***	**CGMCC 3.23546**	**OP082428**	**OP185514**	**OP185521**	**This study**
*Pestalotiopsis telopeae*	CBS 113606	KM199295	KM199498	KM199402	Maharachchikumbura et al., 2014
*Pestalotiopsis telopeae*	CBS 114137	KM199301	KM199559	KM199469	Maharachchikumbura et al., 2014
*Pestalotiopsis telopeae*	CBS 114161	KM199296	KM199500	KM199403	Maharachchikumbura et al., 2014
*Pestalotiopsis terricola*	CBS 141.69	MH554004	MH554438	MH554680	Liu et al., 2019
*Pestalotiopsis thailandica*	MFLUCC 17–1616	MK764285	MK764329	MK764351	Norphanphoun et al., 2019
*Pestalotiopsis thailandica*	MFLUCC 17–1617	MK764286	MK764330	MK764352	Norphanphoun et al., 2019
*Pestalotiopsis trachicarpicola*	OP068	JQ845947	JQ845946	JQ845945	Zhang et al., 2012b
***Pestalotiopsis trachicarpicola***	**CGMCC 3.23547**	**OP082432**	**OP185515**	**OP185522**	**This study**
*Pestalotiopsis unicolor*	MFLUCC 12–0275	JX398998	JX399063	JX399029	Maharachchikumbura et al., 2012
*Pestalotiopsis unicolor*	MFLUCC 12–0276	JX398999	JX399063	JX399030	Maharachchikumbura et al., 2012
*Pestalotiopsis verruculosa*	MFLUCC 12–0274	JX398996	JX399061	-	Maharachchikumbura et al., 2012
*Pestalotiopsis yanglingensis*	LC3067	KX894949	KX895166	KX895281	Liu et al., 2017
*Pestalotiopsis yanglingensis*	LC4553	KX895012	KX895231	KX895345	Liu et al., 2017
*Pseudopestalotiopsis cocos*	CBS 272.29	KM199378	KM199553	KM199467	Maharachchikumbura et al., 2014
*Neopestalotiopsis protearum*	CBS 114178	JN712498	LT853201	KM199463	Maharachchikumbura et al., 2014

The newly generated sequences are in bold. “-” represent the sequences are unavailable.

## Results

### Phylogenetic analyses

**FIGURE 1:**
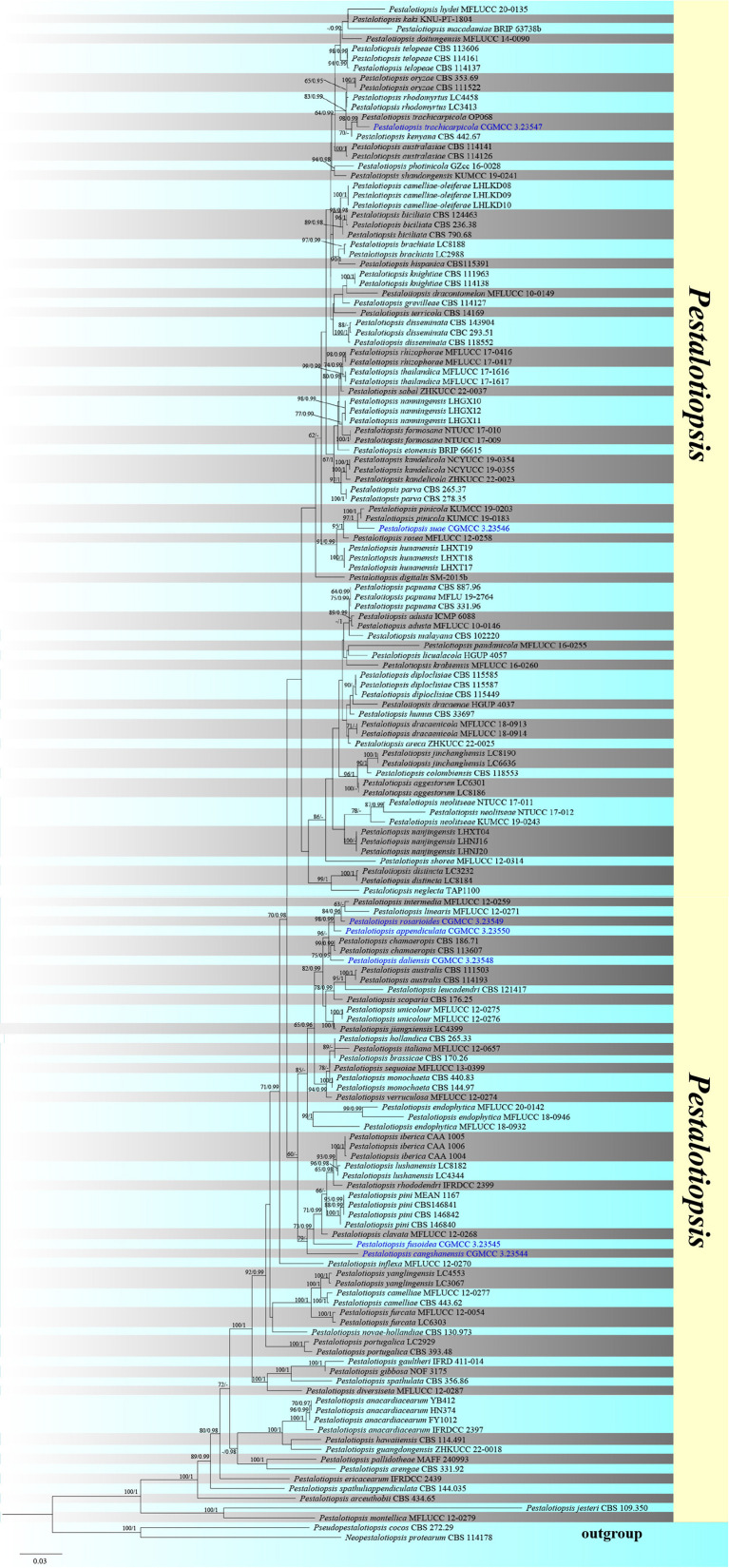
Phylogenetic tree based on RAxML analyses of a combined internal transcribed spacer (ITS), translation elongation factor 1-alpha (TEF1-α), and β-tubulin (TUB) dataset. Bootstrap support values for maximum likelihood ≥60% and Bayesian posterior probabilities ≥0.95 are indicated above the nodes as Maximum likelihood/Posterior probabilitie (ML/PP). The tree is rooted in *Neopestalotiopsis protearum* (CBS 114.178) and *Pseudopestalotiopsis cocos* (CBS 272.29). The new isolates are in blue.

The combined ITS, TEF1-α, and TUB sequence dataset included 154 ingroup taxa and two outgroup taxa (*Neopestalotiopsis protearum* and *Pseudopestalotiopsis cocos*) with 2,160 characters (ITS: 1–538 bp; TEF: 539–1477 bp; TUB: 1478–2160 bp) overall post-alignment, including the gaps. The RAxML and Bayesian analyses of the combined dataset resulted in phylogenetic reconstructions with largely identical topologies and a ML analysis with a final likelihood value of −17482.622268, as shown in Figure 1. The matrix exhibited 993 distinct alignment patterns, with 24.14% undetermined characters or gaps. The estimated base frequencies were as follows: *A* = 0.237874, *C* = 0.294954, *G* = 0.216783, *T* = 0.250389; substitution rates AC = 1.059763, AG = 3.258532, AT = 1.260093, CG = 0.980806, CT = 4.659318, GT = 1.000000; gamma distribution shape parameter α = 0.309168. The bootstrap support values for RAxML greater than 60% and the Bayesian posterior probabilities greater than 0.95 are given at each node (Figure 1).

In the phylogenetic analyses, all new strains were grouped with members of *Pestalotiopsis. Pestalotiopsis rosarioides*, *Pestalotiopsis intermedia*, and *Pestalotiopsis linearis* were grouped together; however, *P. rosarioides* has a separate branch with 84% ML and 0.96 BYPP support. *Pestalotiopsis appendiculata* established a distinct lineage with 98% ML and 0.99 BYPP bootstrap support. *Pestalotiopsis suae* was clustered as a sister taxon to *Pestalotiopsis pinicola* with a significant support (97% ML and 1 BYPP). *Pestalotiopsis daliensis* was clustered as a sister to *Pestalotiopsis chamaeropis* with significant support (75% ML and 0.95 BYPP). *Pestalotiopsis fusoidea*, *Pestalotiopsis cangshanensis*, *Pestalotiopsis pini*, *Pestalotiopsis lushanensis*, *Pestalotiopsis rhododendri*, and *Pestalotiopsis clavate* were grouped together in an independent clade within *Pestalotiopsis*, while *P. fusoidea* and *P. cangshanensis* formed distinct branches. *Pestalotiopsis trachicarpicola* clustered with the ex-type of *P. trachicarpicola* with strong support (98% ML and 0.99 BYPP).

***Pestalotiopsis appendiculata*** D.F. Bao, R. Gu and Z.L. Luo, **sp. nov**.

*MycoBank number*: 845187, Figure 2.

**FIGURE 2:**
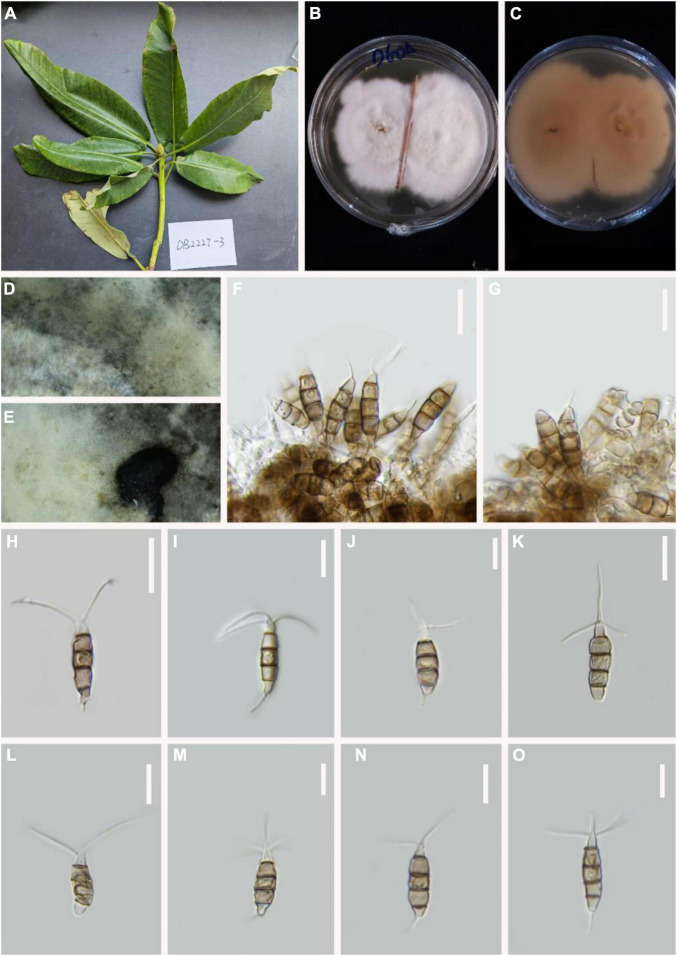
*Pestalotiopsis appendiculata* (KUN-HKAS 124571, holotype). **(A)** Leaves of *Rhododendron decorum*. **(B,C)** Culture on potato dextrose agar (PDA) (upper and lower view). **(D,E)** Conidiomata on PDA. **(F,G)** Conidiophores, conidiogenous cells, and conidia. **(H–O)** Conidia. Scale bars: **(F)** 15 μm, **(G)** 10 μm, **(H)** 15 μm, **(I–O)** 10 μm.

Holotype–KUN-HKAS 124571

Etymology–“appendiculata,” denoting the fungus conidial appendages.

*Endophytic* in fresh *Rhododendron decorum* leaves. **Sexual morph:** Undetermined. **Asexual morph:** *Conidiomata pycnidial* in PDA culture, globose or clavate, aggregated or scattered, semi-immersed to erumpent, gray. There is no evidence of a conidiogenous cell. *Conidia* 19–24 × 5–6 μm ($\bar{x}$ = 21 × 5 μm, *n* = 30), fusoid, ellipsoid, straight to slightly curved, four-septate, slightly constricted at septa. Basal cell 2–4 μm long, conic to obconic with a truncate base, hyaline, verrucose, and thin-walled. Three-median cells doliiform, plicated, 13–15 μm ($\bar{x}$ = 14 μm, *n* = 30). Thin-walled, with a uniform light color on the third cell and the fourth cell relatively darker, the septa darker than the rest of the cells (second cell from the base, 4–6 μm long; third cell 5–6 μm long; fourth cell 4–6 μm long). Apical cell 2–4 μm long, hyaline, subcylindrical, or obconic with a truncated base, thin-walled, slightly rugose. With 2–3 tubular apical appendages arising from the apical crest, unbranched, filiform, 8–15 μm ($\bar{x}$ = 12 μm, *n* = 30). Basal appendage single, tubular, centric, or uncentred, 3–5 μm long.

*Material examined:* China, Yunnan Province, Dali City, Cangshan Mountain, isolated from healthy leaves of *R. decorum* (2489 m, 24.3240°N, 101.0140°E), April 2021, Z.Q. Zhang, D-60 (KUN-HKAS 124571, **holotype**), ex-type culture, CGMCC 3.23550 = KUNCC 22-12405.

*Notes: P. appendiculata*, *P. intermedia*, *P. linearis*, and *P. rosarioides* were grouped in the same clade in the phylogenetic analysis. Comparisons of ITS, TEF-1α, and TUB gene regions of *P. appendiculata* with *P. linearis* (2/538 in ITS, 12/398 in TEF-1α, and 23/450 in TUB), *P. intermedia* (3/537 in ITS, 17/398 in TEF-1α, and 13/479 in TUB), and *P. rosarioides* (3/553 in ITS, 22/553 in TEF-1α, and 22/458 in TUB) indicated significant differences. However, *P. appendiculata* can be distinguished from *P. linearis* and *P. intermedia* with its smaller conidia (Table 2). Moreover, the three-median cells of conidia in *P. appendiculata* are light gray; in contrast, they are brown in *P. linearis*, *P. intermedia*, and *P. rosarioides*. Based on the combined ITS, TEF-1α, and TUB sequence data, our phylogenetic analysis revealed that they are clearly distinct in the phylogram. *P. appendiculata* formed a separate branch with strong support values (98 ML/0.99 PP, Figure 1). Therefore, based on phylogenetic analysis and its morphological characteristics, *P. appendiculata* is introduced as a new species.

**TABLE 2:** Compare the conidia size.

Species	Conidial size	References
*Pestalotiopsis appendiculata*	19–24 × 5–6 μm	**This study**
*P. linearis*	24–33 × 5–6 μm	Maharachchikumbura et al., 2012
*P. intermedia*	24–28 × 6–7 μm	Maharachchikumbura et al., 2012

***Pestalotiopsis cangshanensis*** H.W. Shen, R. Gu and Z.L. Luo, **sp. nov**.

*MycoBank number*: 845188, Figure 3.

**FIGURE 3:**
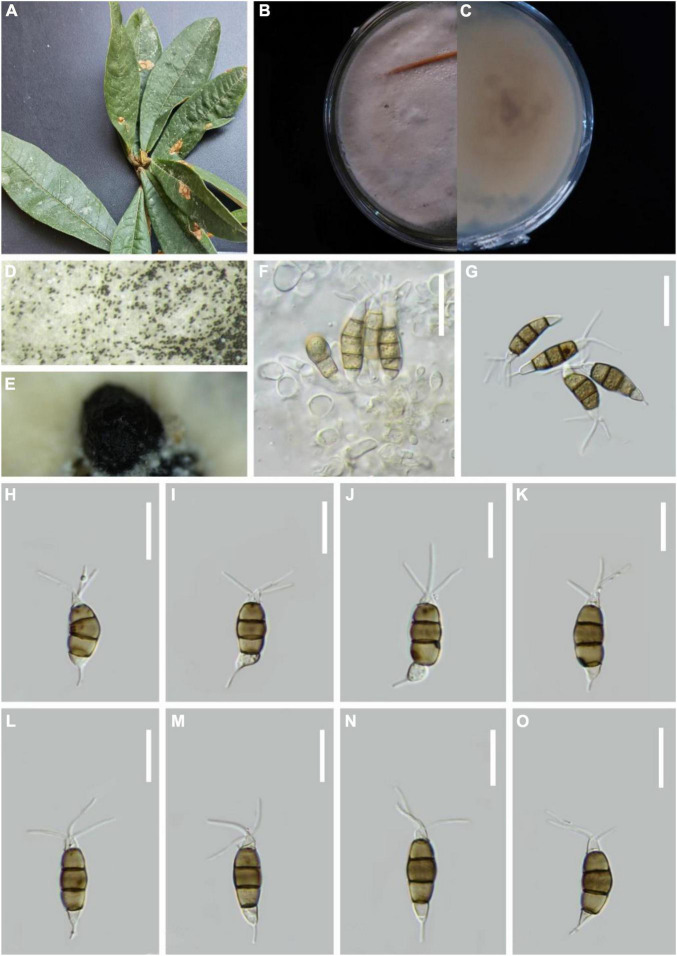
*Pestalotiopsis cangshanensis* (KUN-HKAS 124573, holotype). **(A)** Leaves of *Rhododendron delavayi*. **(B,C)** Culture on potato dextrose agar (PDA) (upper and lower view). **(D,E)** Conidiomata on PDA. **(F)** Conidiophores, conidiogenous cells, and conidia. **(G–O)** Conidia with appendages. Scale bars: **(F–H)** 10 μm, **(I–M)** 15 μm, **(N,O)** 10 μm.

Holotype–KUN-HKAS 124573

Etymology–“cangshanensis,” referring to the Cangshan Mountain, where the species was obtained.

*Endophytic* in fresh *Rhododendron delavayi* leaves. **Sexual morph:** Undetermined. **Asexual morph:** *Conidiomata* pycnidial on PDA, dark brown to black conidial masses, globose, ink-shaped. *Conidiophores* indistinct and typically reduced to conidiogenous cells. *Conidiogenous cells* discrete, subcylindrical to ampulliform, hyaline, sometimes slightly wide at the base, truncated at the apex, 4–24 × 2–4 μm. *Conidia* 18–23 × 6–8 μm ($\bar{x}$ = 19 × 2 μm, *n* = 30), fusoid, straight to slightly curved, four-septate; three-median cells light or dark brown, 10–14 μm long ($\bar{x}$ = 12 μm, *n* = 30; second cell from the base pale-light brown 3–5 μm long; third cell 3–5 μm long; fourth cell 3–5 μm long), wall verruculose, concolourous. Base cell inverted funnel-shaped with a truncated base, 3–4 μm long ($\bar{x}$ = 4 μm), hyaline, thin-walled. Apical cell 4–5 μm long ($\bar{x}$ = 4 μm), hyaline, cylindrical to subcylindrical, thin, and smooth-walled. With three tubular apical appendages 9–19 μm long ($\bar{x}$ = 15 μm, *n* = 30) arising from the apical crest, filiform, unbranched. Basal appendage single, tubular, unbranched, centric, 5–8 μm long ($\bar{x}$ = 7 μm, *n* = 30).

*Material examined*: China, Yunnan Province, Dali City, Cangshan Mountain, isolated from healthy leaves of *R. delavayi* (2489 m, 25.4724°N, 99.5949°E), March 2021, Z.Q. Zhang, D-6 (KUN-HKAS 124573, **holotype**), ex-type culture, CGMCC 3.23544.

*Notes*: *P. cangshanensis*, *P. clavate*, *P. lushanensis*, *P. rhododendri*, and *P. pini* were grouped together. Comparisons of ITS, TEF-1α, and TUB gene regions of *P. cangshanensis* with *P. lushanensis* (2/505 in ITS, 16/932 in TEF-1α, and 12/789 in TUB), *P. pini* (2/605 in ITS, 17/474 in TEF-1α, and 11/792 in TUB), *P. rhododendri* (2/538 in ITS, 17/941 in TEF-1α, and 11/458 in TUB), and *P. clavate* (1/539 in ITS, 10/947 in TEF-1α, and 19/457 in TUB) exhibited significant differences. Morphologically, *P. cangshanensis* has smaller conidia than *P. pini*, *P. clavata*, *P. rhododendri*, and *P. lushanensis* (Table 3). Moreover, *P. cangshanensis* has shorter apical appendages than those of *P. rhododendri* (21–28 vs. 9–19 μm) and *P. lushanensis* (17–26 vs. 9–19 μm). Based on combined ITS, TEF1-α, and TUB sequence data, *P. cangshanensis* is clearly separated in the phylogram as it forms an independent clade. It indicates that *P. cangshanensis* can be introduced as a new species.

**TABLE 3:** Compare the conidia size.

Species	Conidial size	References
*Pestalotiopsis cangshanensis*	18–23 × 6–8 μm	**This study**
*P. pini*	23–25 × 7–8 μm	Silva et al., 2020
*P. clavata*	20–27 × 7–8 μm	Maharachchikumbura et al., 2012
*P. rhododendri*	24–26 × 6–7 μm	Zhang et al., 2013
*P. lushanensis*	20–27 × 8–10 μm	Liu et al., 2017
*P. fusoidea*	22–26 × 6–7 μm	**This study**

***Pestalotiopsis daliensis*** H.W. Shen, R. Gu and Z.L. Luo, **sp. nov**.

*MycoBank number*: 845189, Figure 4.

**FIGURE 4:**
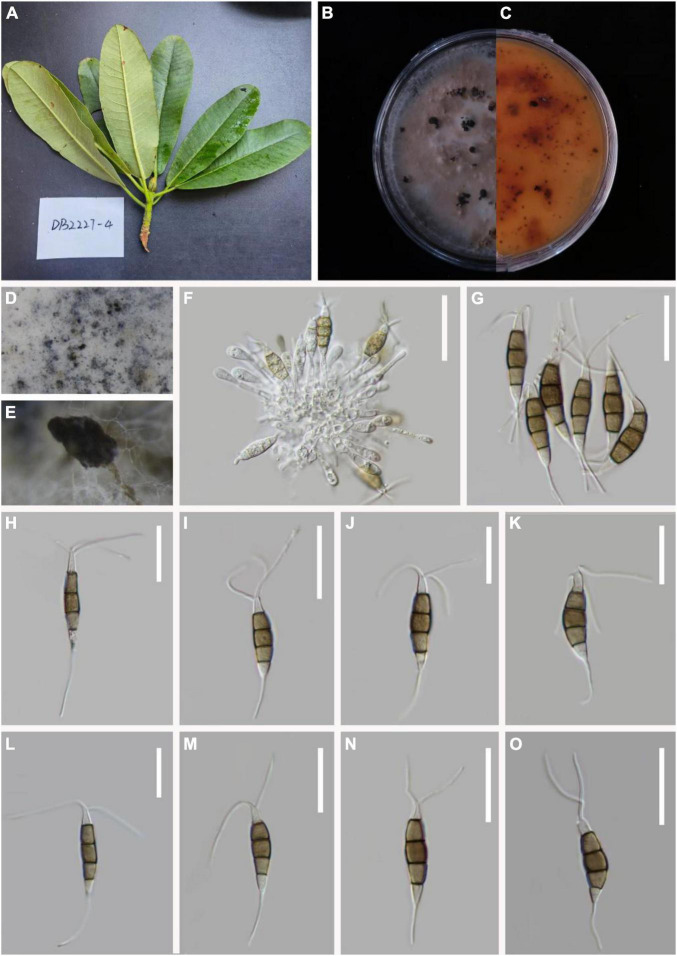
*Pestalotiopsis daliensis* (KUN-HKAS 124576, holotype). **(A)** Leaves of *Rhododendron decorum*. **(B,C)** Culture on potato dextrose agar (PDA) (upper and lower view). **(D,E)** Conidiomata on PDA. **(F)** Conidiophores, conidiogenous cells, and conidia. **(G–O)** Conidia. Scale bars: **(F)** 25 μm, **(G)** 15 μm, and **(H–O)** 10 μm.

Holotype–KUN-HKAS 124576

Etymology–“daliensis,” referring to Dali City, where the species was obtained.

*Endophytic* in fresh *R. decorum* leaves. **Sexual morph:** Undetermined. **Asexual morph:** *Conidiomata* (on PDA) pycnidial, globose to clavate, solitary, exuding globose, dark-brown to black conidial masses. *Conidiophores* are usually indistinct and reduced to conidiogenous cells. *Conidiogenous cells* discrete, subcylindrical to ampulliform, hyaline, sometimes slightly wide at the base, truncated at the apex, 5–19 × 1–3 μm. *Conidia* 23–26 × 4–5 μm ($\bar{x}$ = 25 × 5 μm, *n* = 30), fusoid, ellipsoid, straight to slightly curved, four-septate; basal cell conic with a truncated base, hyaline or light-brown and thin-walled, 4–6 μm long ($\bar{x}$ = 5 μm, *n* = 30). Three-median cells dark, 13–16 μm long ($\bar{x}$ = 15 μm, *n* = 30), wall smooth, concolourous, septa darker than the rest of the cells (second cell from the base pale brown, 4–5 μm long; third cell, 4–5 μm long; fourth cell, 4–6 μm long). Apical cell 4–6 μm long ($\bar{x}$ = 5 μm, *n* = 30), hyaline, subcylindrical, thin-walled, and smooth-walled. With 2–3 tubular apical appendages 13–22 μm long ($\bar{x}$ = 18 μm, *n* = 30), arising from the apical crest, unbranched, filiform. Basal appendage 10–16 μm long ($\bar{x}$ = 13 μm, *n* = 30), single, tubular, unbranched, centric, straight, or slightly curved.

*Material examined*: China, Yunnan Province, Dali City, Cangshan Mountain, isolated from healthy leaves of *R. decorum* (2470 m, 25.5044°N, 100.0542°E), March 2021, Z.Q. Zhang, D-33 (KUN-HKAS 124576, **holotype**), ex-type culture, CGMCC 3.23548 = KUNCC 22-12403.

*Notes*: In the phylogenetic analysis, *P. chamaeropis* and *P. daliensis* are closely associated. Comparisons of ITS, TEF-1α, and TUB gene regions of *P. daliensis* with *P. chamaeropis* (2/599 in ITS, 6/478 in TEF-1α, and 8/774 in TUB) exhibited significant differences. However, the conidia of *P. daliensis* are relatively narrower than *P. chamaeropis* (4–5 vs. 7–9 μm). Moreover, *P. daliensis* has a much longer conidial basal appendage (10–16 vs. 4–9 μm). Hence, *P. daliensis* is introduced as a new species.

***Pestalotiopsis fusoidea*** D.F. Bao, R. Gu and Z.L. Luo, **sp. nov**.

*MycoBank number*: 845190, Figure 5.

**FIGURE 5:**
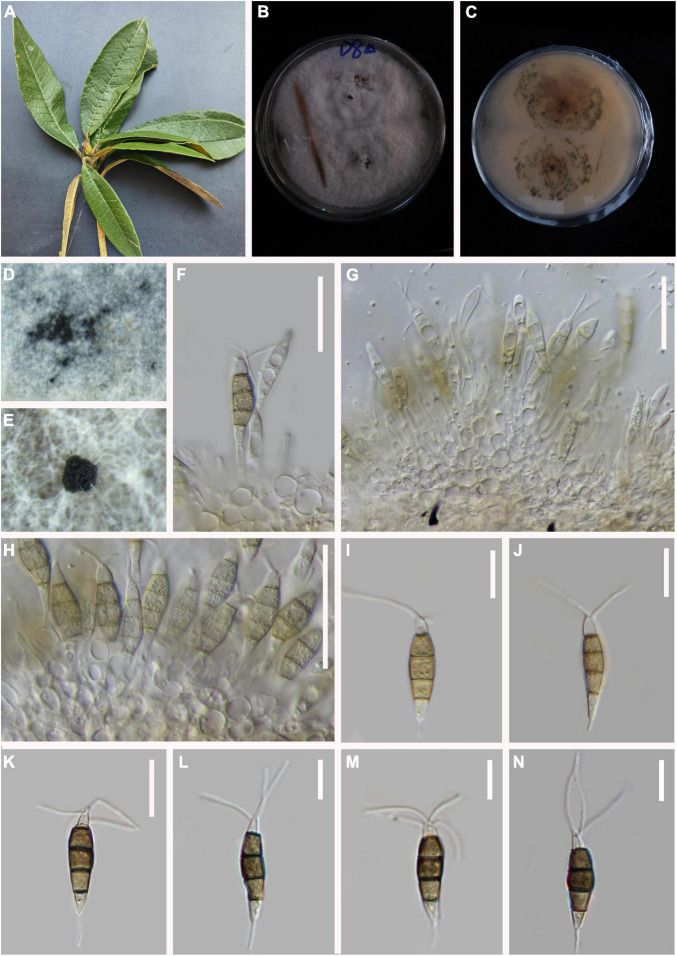
*Pestalotiopsis fusoidea* (KUN-HKAS 124579, holotype). **(A)** Leaves of *Rhododendron delavayi*. **(B,C)** Culture on potato dextrose agar (PDA) (upper and lower view). **(D,E)** Conidiomata on PDA. **(F–H)** Conidiophores, conidiogenous cells, and conidia. **(I–N)** Conidia. Scale bars: **(F–H)** 20 μm, **(I–K)** 15 μm, and **(L–N)** 10 μm.

Holotype–KUN-HKAS 124579

Etymology–“fusoidea,” referring to the fusoid conidia of this fungus.

*Endophytic* in fresh *R. delavayi* leaves. **Sexual morph:** Undetermined. **Asexual morph:** *Colonies* on PDA attaining 15–20 mm in diameter after 7 days at 25°C. Smooth edge, whitish, gregarious. *Conidiomata* formation black droplets, gregarious, reverse pale yellow. *Conidia* aggregate in culture to form black-droplet conidia masses. *Conidiophores* indistinct, typically reduced to conidiogenous cells. *Conidiogenous cells* discrete, subcylindrical to ampulliform, hyaline, and sometimes slightly wide at the base, 5–29 × 2–4 μm. *Conidia* 23–26 × 6–7 ($\bar{x}$ = 25 × 7 μm, *n* = 30), fusoid, four-septate, lightly, curved. Three-median cells 13–18 μm long ($\bar{x}$ = 16 μm, *n* = 30), brown or olive. Some of the third cells are the darkest, second cell 5–6 μm long ($\bar{x}$ = 5 μm, *n* = 30), third cell 4–6 μm long ($\bar{x}$ = 5 μm, *n* = 30), fourth cell 4–6 μm long ($\bar{x}$ = 5 μm, *n* = 30), apical cell 3–4 μm long ($\bar{x}$ = 4 μm, *n* = 30), hyaline, cylindrical to subcylindrical, with 2–4 (or mostly 3) tubular apical appendages 7–11 μm long ($\bar{x}$ = 8 μm, *n* = 30) long arising from the apical crest, filiform. The base cell is an inverted triangle 4–6 μm long ($\bar{x}$ = 4 μm, *n* = 30), with light brown or almost transparent hyaline. Basal appendage single, tubular, unbranched, centric, vertical, or curved, 4–6 μm long ($\bar{x}$ = 6 μm, *n* = 30).

*Material examined*: China, Yunnan Province, Dali City, Cangshan Mountain, isolated from healthy leaves of *R. delavayi* (2716 m, 25.5032°N, 100.4265°E), March 2021, Z.Q. Zhang, D-8 (KUN-HKAS 124579, **holotype**), ex-type culture CGMCC 3.23545 = KUNCC 22-12401.

*Notes*: Phylogenetically, *P. fusoidea* has a close with *P. clavata*, *P. lushanensis*, *P. rhododendri*, and *P. pini*. Comparisons of ITS, TEF-1α, and TUB gene regions of *P. fusoidea* with *P. lushanensis* (2/505 in ITS, 16/932 in TEF-1α, and 12/789 in TUB), *P. rhododendri* (2/538 in ITS, 13/941 in TEF-1α, and 11/458 in TUB), *P. clavate* (9/539 in ITS, 14/947 in TEF-1α, and 11/457 in TUB), *P. pini* (2/571 in ITS, 17/512 in TEF-1α, and 11/514 in TUB) exhibited significant differences. However, *P. fusoidea* has shorter apical appendages than *P. pini* (7–11 vs. 18–20 μm), *P. clavate* (7–11 vs. 20–25 μm), or *P. rhododendri* (7–11 vs. 21–29 μm). *P. fusoidea* has smaller conidia than *P. lushanensis* (23–26 × 6–7 vs. 18–23 × 6–8 μm). Based on combined ITS, TEF1-α, and TUB sequence data, *P. fusoidea* are apparently separated in the phylogram, forming a separate clade. It indicates that *P. fusoidea* can be introduced as a new species.

***Pestalotiopsis rosarioides*** H.W. Shen, R. Gu and Z.L. Luo, **sp. nov**.

*MycoBank number*: 845191, Figure 6.

**FIGURE 6:**
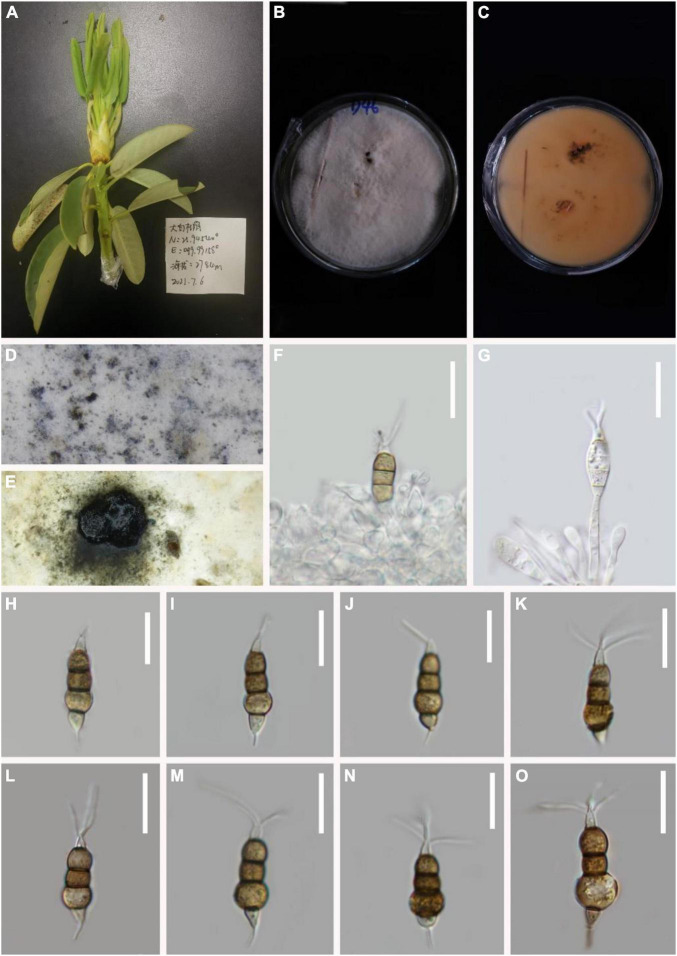
*Pestalotiopsis rosarioides* (KUN-HKAS 124574, holotype). **(A)** Leaves of *Rhododendron decorum*. **(B,C)** Culture on potato dextrose agar (PDA) (upper and lower view). **(D,E)** Conidiomata on PDA. **(F,G)** Conidiophores, conidiogenous cells, and conidia. **(H–O)** Conidia. Scale bars: **(F–O)** 15 μm.

Holotype–KUN-HKAS 124574

Etymology–“rosarioides,” referring to the rosary-like enlargement of the second and fourth cells of this fungus.

*Endophytic* in fresh *R. decorum* leaves. **Sexual morph:** Undetermined. **Asexual morph:** *Conidiomata* (on PDA) pycnidial, globose to clavate, solitary, exuding globose, dark-brown to black conidial masses. *Conidiogenous cells* are not obvious. *Conidia* 22–25 × 6–7 μm ($\bar{x}$ = 23 × 7 μm, *n* = 30), fusoid, ellipsoid, rosary, straight to slightly curved, four-septate. Basal cell conic with a truncated base, hyaline or light brown, and thin-walled, 4–5 μm long ($\bar{x}$ = 5 μm, *n* = 30). Three-median cells dark, 16–18 μm long ($\bar{x}$ = 17 μm, *n* = 30), smooth wall, concolourous, septa darker than the rest of the cells (second cell from the base pale brown and enlarged, 4–5 μm long; third cell 4–5 μm long; fourth cell expands to 3–6 μm long). Apical cell 4–7 ($\bar{x}$ = 5 μm, *n* = 30) long, hyaline, subcylindrical, smooth-walled. With 1–3 tubular apical appendages 5–9 μm long ($\bar{x}$ = 7 μm, *n* = 30) arising from the apical crest, unbranched, filiform. Basal appendage 4–5 μm long ($\bar{x}$ = 4 μm, *n* = 30), single, tubular, unbranched, centric.

*Material examined:* China, Yunnan Province, Dali City, Cangshan Mountain, isolated from healthy leaves of *R. decorum* (2784 m, 25.9454°N, 99.9915°E), July 2021, Z.Q. Zhang, D-46 (KUN-HKAS 124574, **holotype**), ex-type culture, CGMCC 3.23549 = KUNCC 22-12404.

*Notes:* From the phylogenetic analysis, *P. intermedia*, *P. linearis*, and *P. rosarioides* clustered within the same clade. Comparisons of ITS, TEF-1α, and TUB gene regions of *P. rosarioides* with *P. intermedia* (2/537 in ITS, 2/946 in TEF-1α, and 9/479 in TUB), and *P. linearis* (2/538 in ITS, 4/946 in TEF-1α, and 12/450 in TUB) exhibited significant differences. However, the second and fourth conidial cells of *P. rosarioides* are enlarged, which is distinct from other species in the genus. Moreover, *P. rosarioides* has much shorter apical appendages than *P. linearis* and *P. intermedia* (5–9 μm in *P. rosarioides* vs. 10–20 μm in *P. linearis* and 10–28 μm in *P. intermedia*). Furthermore, phylogenetic analysis indicates that *P. rosarioides* forms a distinct lineage within the clade (Figure 1), supporting it as a new species.

***Pestalotiopsis suae*** H.W. Shen, R. Gu and Z.L. Luo, **sp. nov**.

*MycoBank number*: 845192, Figure 7.

**FIGURE 7:**
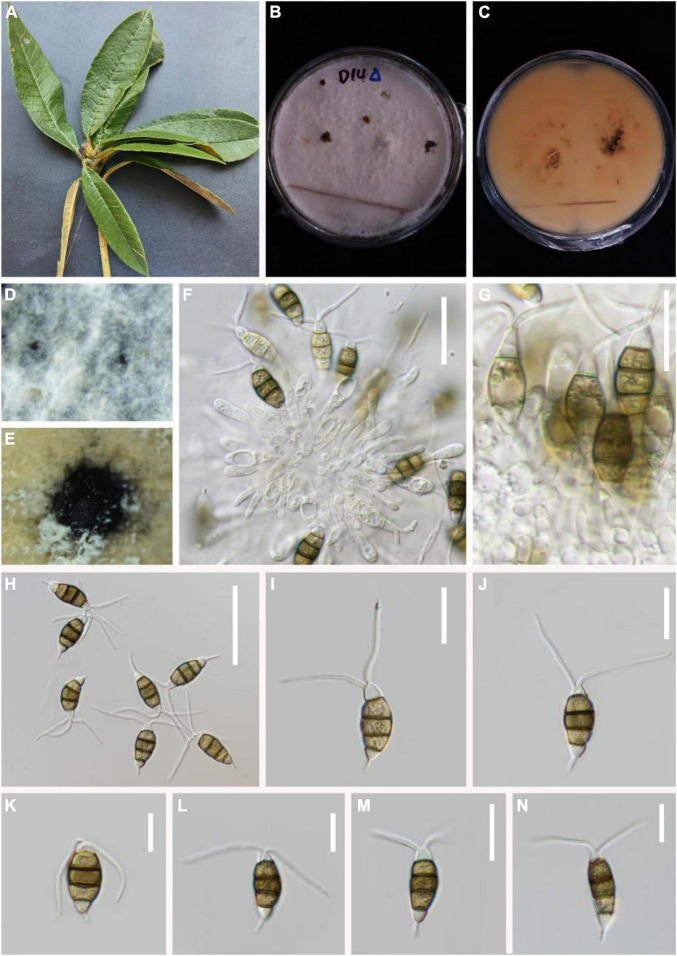
*Pestalotiopsis suae* (KUN-HKAS 124578, holotype). **(A)** Leaves of *Rhododendron delavayi*. **(B,C)** Culture on potato dextrose agar (PDA) (upper and lower view). **(D,E)** Conidiomata on PDA. **(F,G)** Conidiophores, conidiogenous cells, and conidia. **(H–N)** Conidia with appendages. Scale bars: **(F)** 20 μm, **(G–J)** 15 μm, **(K–N)** 10 μm.

Holotype–KUN-HKAS 124578

Etymology–“suae” in memory of the Chinese mycologist Prof. Hong-Yan Su, who kindly helped the authors in many ways and sadly passed away on 3 May 2022 during the preparation of the current article.

*Endophytic* in fresh *R. delavayi* leaves. **Sexual morph:** Undetermined. **Asexual morph:** *Conidia* aggregate in culture to form black-droplet conidia masses. *Conidiophores* indistinct and typically reduced to conidiogenous cells. *Conidiogenous cells* discrete, subcylindrical to ampulliform, hyaline, sometimes slightly wide at the base 5–19 × 1–3 μm. *Conidia* 17–24 × 4–8 μm ($\bar{x}$ = 23 × 7 μm, *n* = 30), fusoid, four-septate. A distinct dark separation exists between each cell, lightly curved, including three-median cells 7–16 μm long ($\bar{x}$ = 17 μm, *n* = 30), brown or olive, with the third cell darker. Apical cell 3–4 μm long ($\bar{x}$ = 4 μm, *n* = 30), hyaline, cylindrical to subcylindrical, with 2–3 tubular apical appendages (mostly 2), 7–11 μm long ($\bar{x}$ = 8 μm, *n* = 30), arising from the apical crest, filiform. Second cell 5–6 μm long ($\bar{x}$ = 5 μm, *n* = 30); third cell 4–6 μm long ($\bar{x}$ = 5 μm, *n* = 30); fourth cell 4–6 μm long ($\bar{x}$ = 5 μm, *n* = 30). Base cell is an inverted triangle, 4–6 μm long ($\bar{x}$ = 4 μm, *n* = 30), light brown or almost transparent hyaline. Basal appendage single, tubular, unbranched, centric, vertical, or curved, 4–6 μm long ($\bar{x}$ = 6 μm, *n* = 30).

*Material examined*: China, Yunnan Province, Dali City, Cangshan Mountain, isolated from healthy leaves of *R. delavayi* (2489 m, 25.4659°N, 99.5829°E), March 2021, Z.Q. Zhang, D-14 (KUN-HKAS 124578, **holotype**), ex-type culture, CGMCC 3.23546 = KUNCC 22-12402.

*Notes*: Based on phylogenetic analysis, the newly discovered *P. suae* is closely related to *P. rosea* and *P. pinicola*. Comparisons of ITS, TEF-1α, and TUB gene regions of *P. suae* with *P. rosea* (3/539 in ITS, 13/943 in TEF-1α, and 9/453 in TUB), and *P. pinicola* (10/608 in ITS, 9/467 in TEF-1α, and 5/409 in TUB) exhibited significant differences. However, *P. suae* is different from *P. rosea* due to its brown conidia, while the conidia of *P. rosea* are slightly red. *P. suae* can be distinguished from *P. pinicola* due to its size of apical and basal appendages; *P. suae* has shorter apical appendages (5–17 vs. 7–11 μm) and longer basal appendages (2–7 vs. 4–6 μm).

**FIGURE 8:**
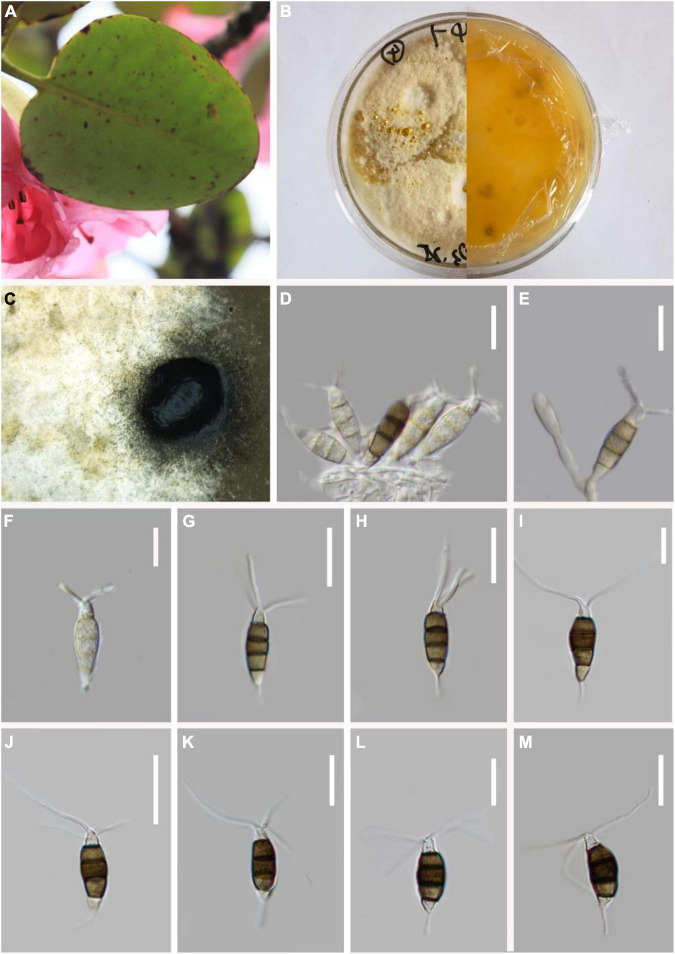
*Pestalotiopsis trachicarpicola* (KUN-HKAS 124577). **(A)** Leaves of *Rhododendron cyanocarpum*. **(B)** Culture on potato dextrose agar (PDA) (upper and lower view). **(C)** Conidiomata on PDA. **(D,E)** Conidiophores, conidiogenous cells, and conidia. **(F–M)** Conidia. Scale bars: **(D–M)** 10 μm.

***Pestalotiopsis trachicarpicola*** Y.M. Zhang and K.D. Hyde, *Cryptog. Mycol.* 33(3):311–318 (2012). Figure 8.

*Endophytic* in fresh *Rhododendron cyanocarpum* leaves. **Sexual morph:** Undetermined. **Asexual morph:** *Conidiomata* pycnidial in culture on PDA, globose or clavate, aggregated or scattered, semi-immersed to erumpent, dark-brown to black. *Conidiogenous cell* is not obvious. Conidiophores are usually indistinct and reduced to conidiogenous cells. *Conidia* 19–23 × 5–6 μm ($\bar{x}$ = 21 × 5 μm, *n* = 30), fusoid, ellipsoid, straight to slightly curved, four-septate, slightly constricted at the septa. Basal cell conic to obconic with a truncated base, hyaline, verruculose, and thin-walled, 2–4 μm long. Three median cells doliiform, 11–14 μm ($\bar{x}$ = 12 μm, *n* = 30). Wall thin, color uniform (light or dark brown), septa darker than the rest of the cells, and the conidium constructed at the septum (second cell from the base, 4–5 μm long; third cell, 5–6 μm long; fourth cell, 4–6 μm long). Apical cell 2–4 μm long, hyaline, subcylindrical, or obconic with a truncated base, thin-walled, slightly rugose. With 2–3 tubular apical appendages arising from the apical crest, unbranched, filiform, 13–23 μm ($\bar{x}$ = 18 μm, *n* = 30). Basal appendage single, tubular, centric, or uncentred, 4–8 μm long.

*Material examined*: China, Yunnan Province, Dali City, Cangshan Mountain, isolated from healthy leaves of *R. cyanocarpum*, March 2021, Z.Q. Zhang, D-20 (KUN-HKAS 124577), living culture, CGMCC 3.23547.

*Notes*: Based on the phylogenetic analysis, *P. trachicarpicola* can be grouped with *P. trachicarpicola* (OP068) with strong support (98% ML and 0.99 BYPP). The morphologies of the two species are identical. For the first time, *P. trachicarpicola* is isolated from *Rhododendron*.

## Discussion

Many fungal groups, such as *Aspergillus*, *Ceratobasidium*, *Fusarium*, *Neocosmospora*, *Pestalotiopsis*, *Pyrenochaeta*, *Russula*, *Serendipita*, *Thanatephorus*, and *Trichoderma* have been reported as endophytic fungi (Fu et al., 2022). As an ornamental plant, *Rhododendron* has achieved worldwide recognition (Cox and Cox, 1997). Recent research has isolated fungi from the leaf spots, mycorrhizae, and rhizosphere of *Rhododendron* (Medeiros et al., 2022). However, few studies have been conducted on the endophytic fungi of *Rhododendron*. Yunnan Province is one of the world’s most significant distribution centers for *Rhododendron* (Tian et al., 2011). There are 61 species of *Rhododendron* in Cangshan Mountain, Yunnan Province, China (Zhang et al., 2021). Our investigation indicates high diversity of *Pestalotiopsis* species in *Rhododendron*. However, the current study collected the leaves of three *Rhododendron* species only. In future research, it is possible to expand the survey area and collect additional *Rhododendron* leaves to obtain more endophytic fungal resources.

Previous studies mentioned that the color intensities of the median conidial cell, differences in the size variation of conidia, and the presence or absence of basal appendages can be used as additional taxonomic characteristics for distinguishing *Pestalotiopsis* species (Jeewon et al., 2003; Liu et al., 2017). Apical appendage characteristics, such as branching pattern, number, and attachment position, are also useful at the species level, but not at the generic level of a generic character (Crous et al., 2012). Therefore, it was proposed to implement additional morphological characteristics for species identification. ITS, TUB, and TEF1-α gene sequences can be combined to provide greater resolution for *Pestalotiopsis* (Li et al., 2021). In our phylogenetic tree, branch lengths of *Pestalotiopsis cangshanensis*, *P. fusoidea*, and some other species in this genus were notably short and the support values were relatively low. Further studies of *Pestalotiopsis* are, therefore, required to reveal whether the less informative loci lead to the poorly resolved phylogram.

## Data availability statement

The datasets presented in this study can be found in online repositories. The names of the repository/repositories and accession number(s) can be found below: https://www.ncbi.nlm.nih.gov/genbank/ (OP082426, OP185510, OP185517, OP082429, OP185511, OP185518, OP082431, OP185509, OP185516, OP082427, OP185512, OP185519, OP082430, OP185513, OP185520, OP082428, OP185514, OP185521, OP082432, OP185515, and OP185522).

## Author contributions

RG conducted the experiments, analyzed the data, and wrote the manuscript. D-FB, Z-LL, and H-WS revised the manuscript. H-WS planned the experiments and analyzed the data. Z-LL and X-JS planned and funded the experiments. X-JS and Y-XL helped the experiments. All authors revised the manuscript.

## Footnotes

1
https://www.mycobank.org
